# Comparative Evaluation of Heavy Metal Concentrations in Residents of Abandoned Metal Mines

**DOI:** 10.3390/ijerph17176280

**Published:** 2020-08-28

**Authors:** Jeong-wook Seo, Young-seoub Hong

**Affiliations:** 1 Environmental Health Center, Dong-A University, Busan 49201, Korea; jwseo@dau.ac.kr; 2 Department of Preventive Medicine, Dong-A University, Busan 49201, Korea

**Keywords:** abandoned mine, lead, cadmium

## Abstract

This study compares the heavy metal exposure levels of the population of abandoned metal mines, with high risks of environmental pollution and health effects. We used data from a two-stage abandoned metal mines survey (AMS, n = 4467). The Korea National Health and Nutrition Examination Survey (KNHANES) and the Korea National Environmental Health Survey (KNEHS) were used as general population data. Based on the sex and age distribution in the AMS, a simple random sampling was performed, so that the two datasets had the same distribution (KNHANES n = 1815, KNEHS n = 2328). Blood lead concentrations were slightly higher in the AMS than in KNEHS. Blood cadmium concentrations were similar between the two groups. However, the difference in urine cadmium concentrations was pronounced and statistically significant. Moreover, 30.6% of the AMS data for urine cadmium concentration exceeded the 95th percentile of the KNEHS data. The concentration of lead and cadmium in the residents of the abandoned metal mines, i.e., the vulnerable regions, was higher than that in the general population. It is necessary to monitor and manage the vulnerable regions via a more active and extensive survey system.

## 1. Introduction

Abandoned metal mines are classified as the most vulnerable regions with the highest level of contamination among the soil contamination risk regions in Korea [1]. Soils are contaminated by pollutants such as heavy metals from abandoned metal mines. This affects nearby rivers and the groundwater, ultimately increasing the risk of human exposure and health problems, which poses the need for special attention on a national level and continuous management.

Biomonitoring projects in Korea can be classified into the monitoring of the general public and that of the population in vulnerable regions. The Environmental Health Act of Korea states that through the biomonitoring of residents in vulnerable regions, the effect and damage from environmental hazards should be investigated, taking every measure needed to protect public health. In particular, it is necessary to designate abandoned metal mines, industrial complexes, and thermal power plants as regions vulnerable to environmental pollution and to monitor and manage the health status of residents in the applicable areas. In this regard, the Korea National Institute of Environmental Research (KNIER) conducts biomonitoring projects, such as the “Health Effects Survey of Abandoned Metal Mines (AMS)”, the “Environmental pollution exposure and health impact monitoring project for the residents in the national industrial complexes”, and the “Resident health impact survey in regions around coal-fired power stations”. These surveys are primarily aimed at identifying the level of exposure to hazardous substances in the environment, the exposure pathways, and the level of exposure to residents in the affected area, focusing on contaminants in the vulnerable regions as well as their correlations with health effects. Abandoned metal mines are areas of concern due to their high risk of soil contamination by various types of hazardous metals. The regions show relatively higher pollution levels of lead and cadmium as compared to other heavy metals, and the possibility of health effects from long-term low-level exposure to heavy metals on residents in the affected area is likely to be high.

The national biomonitoring projects for the general public include the Korea National Health and Nutrition Examination Survey (KNHANES) of the Korea Centers for Disease Control and Prevention (KCDC), and the Korea National Environmental Health Survey (KNEHS) by the Korea National Institute of Environmental Research (KNIER). These monitoring projects are both designed to present values representative of Koreans in terms of lead, cadmium, mercury, and arsenic exposure levels. This study compares the heavy metal exposure levels of the population of abandoned metal mines and those of the general population by using a two-stage AMS conducted at 104 places of abandoned metal mines with high risk of environmental pollution and health effects. 

## 2. Materials and Methods 

### 2.1. Study Subjects and Materials

#### 2.1.1. AMS

**Figure 1:**
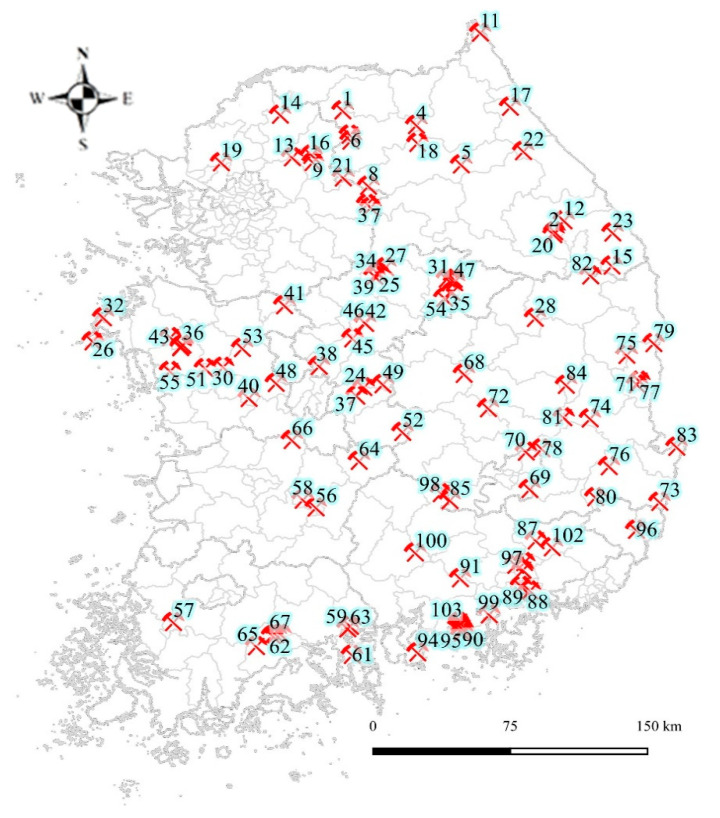
Locations of the 103 abandoned metal mine areas.

The Ministry of Environment (KMOE) reported the results of the outlook survey and the detailed survey on soil and water system pollution levels of 857 abandoned metal mines from 1996 to 2005. Subsequently, preliminary surveys were carried out on 358 abandoned metal mines with risks of adverse health effects, and the overall risk was quantified according to the results of environmental pollution and health impact assessment from these surveys. The first stage AMS survey (2008–2011) was conducted for 38 places corresponding to the top 10%, and the second stage survey (2013–2017) was conducted for 103 mines of the top 10–40%(Figure 1). The second-stage AMS divided the whole country into five regions, and a total of 4500 people were surveyed for five years, in accordance with standardized tools and guidelines. In addition to the questionnaire on the area of residence and subject characteristics, an analysis of heavy metals, such as on the contents of blood lead, blood cadmium, urine cadmium, and urine arsenic, was carried out. The types, size, period of operation, and closure of abandoned metal mines; the presence and characteristics of the mining pollution treatment; and the degree of contamination may differ between the abandoned mines but were not included as impact factor variables in this study. However, compared with the general population, they can be classified into the direct or indirect heavy metal exposure groups due to the shared characteristics of abandoned metal mines. The study subjects were residents who lived within 5 km of the pit head and tailings of each abandoned metal mine. All procedures related to biological sample collection and heavy metal analysis of the AMS followed KNIER’s standard guidelines [2,3].

#### 2.1.2. KNHANES

Similar to U.S. NHANES, KNHANES is a survey with a rolling sampling design, which divides national samples into three independent rolling samples surveyed over three years. These are probabilistic samples representing the whole country and designed such that their characteristics are the same. Therefore, it is possible to produce statistics on a national level with each rolling sample, which can be used as total population survey data when three years of survey are combined. KNHANES consists of four categories: health status survey, public health awareness behavior survey, physical examination survey, and nutrition survey. In this study, a rolling sampling design was applied and data from surveys in 2008–2013, 2016, and 2017 (KNHANES IV-2, IV-3, V, VI-1, VII-1, VII-2, n = 16,872) were used, which include the period of the second-stage AMS and feature comparable demographic and lifestyle characteristics. All procedures related to the KNHANES’s biological sample collection and heavy metal analysis were in compliance with KNHANES’s standard guidelines [4].

#### 2.1.3. KNEHS

KNEHS is a survey with a rolling sampling design, focused on identifying human exposure levels to environmental hazards, similar to KNHANES. KNEHS is categorized into three areas: environmental exposure survey, environmentally hazardous substance analysis, and physical examination. This study used data from stages 1 and 2 (2009–2014, n = 16,576) and included comparable demographic and lifestyle characteristics. All procedures related to the KNEHS’ collection of biological samples and heavy metal analysis were in compliance with KNIER’s standard guidelines [3,5].

### 2.2. Statistical Analysis

To directly compare the distribution of heavy metal concentrations according to the data source, simple random resampling was performed on the general population data, KNHANES and KNEHS, at the same ratio based on the sex and age ratio of the AMS, i.e., the exposed group data. At this time, 99 resampled samples were composed, and the sample corresponding to the median value was selected based on the geometric mean of the blood and urine cadmium concentration. As for the blood and urine heavy metals, the center position was presented with a skewed distribution (skewness > 0), and the geometric mean and 95% confidence intervals were presented with interval estimators. The least square mean of each factor was tested to compare the concentration level according to the characteristics of subjects in the group and for comparison between groups. In this case, Bonferroni correction was performed. The AMS excess ratio is given with reference to the 95th percentile concentration levels of the general populations of the KNHANES and KNEHS data. For all estimators and tests, SAS (Version 9.4, SAS Institute, Cary, NC, USA) was used, and the tests were performed under the significance level of 5%.

### 2.3. Ethics Statement

A data collection agreement (for the questionnaire and blood samples) was secured from all participants after providing them with a full explanation of the purpose and procedure of the study. We then arranged for the personal information collected and the results of specimen analysis to be available to the participants. The present study received approvals from the Chung-Ang University Institutional Review Board (IRB no. 1041078-201805-HRBR-103-01).

## 3. Results

### 3.1. Heavy Metal Concentration Distribution

The distribution of heavy metals by data source is shown in Table 1.

The geometric mean (95% confidence interval) of the blood lead concentration in AMS was 2.28 (2.25–2.31) μg/dL, somewhat higher than 2.21 (2.16–2.25) μg/dL of KNHANES and 2.11 (2.14–2.23) μg/dL of KNEHS. No significant difference was found in the high concentration distribution above the 95th percentile. In the case of blood cadmium, the AMS result was higher at 1.43 (1.40–1.45) μg/L as compared to that of KNHANES at 1.24 (1.22–1.27) μg/L, and the 95th percentile was 3.80 μg/L and 2.73 μg/L, respectively, showing a significant difference. In particular, the urine cadmium concentration level of 1.67 (1.62–1.73) µg/g cr of AMS was higher than that of KNEHS with 0.82 (0.80–0.85) µg/g cr. The 95th percentile differed more than twofold, with 5.81 and 2.49 μg/g cr, respectively. The urine arsenic concentration was found to be higher in KNEHS data with 42.09 (39.65–44.68) μg/L compared to 30.32 (29.50–31.17) μg/L in AMS, but at the higher concentration range above the 99th percentile, the AMS value was higher.

### 3.2. Comparison of Blood Lead Concentration

The comparison results of the distribution of AMS characteristics and blood lead concentrations are shown in Table 2.

In this study, when we examined the characteristics of the demographic and sociological distribution of 4478 AMS subjects, who were analyzed for at least one type of heavy metal, the proportion of females was 60.7%, higher than that of males, and those in their 70s or older had a very high proportion at 52.7%, indicating a high proportion of an aged population compared to the comparatively lower age group of 19–39 years (1.1%) and 40–49 years (3.9%).

The geometric mean (95% confidence interval) of the blood lead concentration adjusted for sex, age, smoking status, drinking status, monthly household income, and year of survey was 2.39 (2.30–2.48) μg/dL for AMS, which is somewhat higher than that of KNHANES, 2.24 (2.14–2.35) μg/dL, and showed a statistically significant difference to that of KNEHS, with 1.92 (1.83–2.00) μg/dL (*p* < 0.001).

Men had significantly higher lead concentrations in the AMS data as compared to women (men: 2.64 (2.54–2.74) μg/dL women: 2.16 (2.07–2.25) μg/dL, *p* < 0.001). As for the age distribution, there was a significant increase in the lead concentration as the age increased from the young age group to the group in their 50s, and then the concentration decreased again beyond that age (19–39 years: 1.88 (1.68–2.11) μg/dL, 40s: 2.38 (2.23–2.54) μg/dL, 50s: 2.68 (2.58–2.78) μg/dL, 60s: 2.61 (2.52–2.71) μg/dL, 70s or older: 2.47 (2.38–2.56) μg/dL, *p* < 0.001). This pattern of lead concentrations by sex and age was also confirmed in the KNHANES and KNEHS results. However, the lead concentrations of the 19–39 years group in KNHANES (1.96 (1.64–2.34) μg/dL) were higher than that of the same group in AMS, indicating that the spread of concentrations by age group is larger in the AMS data. Seoul, Gyeonggi, and Gangwon Province in the AMS data showed relatively higher lead concentrations than other regions (Seoul, Gyeonggi, Gangwon: 2.74 (2.62–2.86) μg/dL, Chungcheong: 2.55 (2.45–2.66) μg/dL, Honam: 2.27 (2.16–2.38) μg/dL, Youngnam: 2.25 (2.16–2.35) μg/dL, *p* < 0.001). These results differed from those of KNHANES and KNEHS. This could be attributed to the fact that the abandoned metal mines in Seoul, Busan, Incheon, Gwangju, Daejeon, Gyeonggi, and Jeju were not investigated in the AMS. In addition, there are limitations with respect to the separation of the regional characteristics and effect of exposure potential according to the degree of heavy metal contamination of the individual abandoned metal mines.

In terms of lifestyle, lead concentration levels increased in the order non-smoker, i.e., people who never smoked, past smoker, and current smoker (current smoker: 2.54 (2.42–2.66) μg/dL, past smoker: 2.38 (2.27–2.50) μg/dL, non-smoker: 2.25 (2.17–2.34) μg/dL, *p* < 0.001). Similarly, the lead concentrations of current drinkers were relatively higher than those of former drinkers and those who have never been drinking (current drinker: 2.56 (2.47–2.66) μg/dL, former drinker: 2.31 (2.19–2.43) μg/dL, non-drinker (never drank): 2.25 (2.17–2.34) μg/dL, *p* < 0.001). The same trend was observed in the KNHANES and KNEHS data. 

There was no clear trend in the lead concentrations in AMS with respect to an increase or decrease of the monthly household income (less than 1 million won: 2.37 (2.29–2.46) µg/dL, between 1 and less than 2 million won: 2.45 (2.34–2.57) µg/dL, between 2 and less than 3 million won: 2.26 (2.12–2.40) μg/dL, more than 3 million won: 2.47 (2.32–2.63) μg/dL, *p* = 0.034). On the other hand, KNHANES and KNEHS showed a clear trend of decreasing lead concentrations as the monthly household income increased. In terms of the monthly household income, eating habits according to regional characteristics may act as a confounder. In the case of AMS, most survey results are obtained from suburban areas.

In the AMS survey, the distance from the mine to the residential area and the work experience in the mine were examined. As the distance from the mine increased, lead concentration levels tended to decrease slightly (less than 0.5 km: 2.29 (2.19–2.39) µg/dL, between 0.5 and less than 1.0 km: 2.29 (2.20–2.38) µg/dL, between 1.0 and less than 1.5 km: 2.22 (2.13–2.31) μg/dL, between 1.5 and less than 3.0 km: 2.30 (2.21–2.41) μg/dL, 3.0 km or more: 2.13 (2.02–2.25) μg/dL, *p* = 0.011). Higher lead concentration levels were observed in subjects with past mining experiences (with experience: 2.31 (2.21–2.42) μg/dL, no experience: 2.26 (2.19–2.34) μg/dL, *p* = 0.249).

### 3.3. Comparison of Blood Cadmium Concentration

Table 3 shows the comparison results of blood cadmium concentrations adjusted for sex, age, smoking status, drinking status, monthly household income, and year of survey.

For AMS, the concentration was 1.19 (1.13–1.25) μg/L, which was very similar to that of the KNHANES data, 1.22 (1.15–1.29) μg/L (*p* = 0.564).

Women showed significantly higher concentrations than men (men: 0.98 (0.94–1.03) μg/L, women: 1.43 (1.35–1.52) μg/L, *p* < 0.001). A clear tendency for increased cadmium concentrations in older age groups was observed (19–39: 0.79 (0.69–0.92) μg/L, 40s: 1.14 (1.05–1.24) μg/L, 50s: 1.30 (1.23–1.36) μg)/L, 60s: 1.38 (1.31–1.45) μg/L, 70s or more: 1.46 (1.39–1.53) μg/L, *p* < 0.001). The cadmium concentration patterns by sex and age were also the same in the KNHANES results. The southern regions of Yeongnam and Honam of the AMS data showed relatively higher cadmium concentrations than the other regions (Seoul, Gyeonggi, Gangwon: 0.93 (0.88–0.99) μg/L, Chungcheong: 1.19 (1.13–1.25) μg/L, Honam: 1.20 (1.13–1.28) μg/L, Yeongnam: 1.48 (1.40–1.56) μg/L, *p* < 0.001). The distribution pattern was similar to that of KNHANES. 

Smokers had significantly higher cadmium concentrations than past smokers and non-smokers (current smoker: 1.42 (1.34–1.52) μg/L, past smoker: 1.13 (1.06–1.20) μg/L, non-smoker: 1.04 (0.99–1.10) μg/L, *p* < 0.001). There was no characteristic tendency according to current drinking status (current drinker: 1.20 (1.14–1.26) μg/L, former drinker: 1.14 (1.07–1.22) μg/L, non-drinker: 1.22 (1.16–1.29) μg/L, *p* = 0.023). As the monthly household income decreased, the cadmium concentrations increased significantly (less than 1 million won: 1.25 (1.19–1.31) μg/L, between 1 and less than 2 million won: 1.17 (1.11–1.25) μg/L, between 2 and less than 3 million won: 1.16 (1.07–1.25) μg/L, more than 3 million won: 1.18 (1.09–1.27) μg/L, *p* = 0.025). This trend was also observed in the KNHANES results. No linear trends were identified with the distance from the mine (less than 0.5 km: 1.20 (1.13–1.27) µg/L, between 0.5 and less than 1.0 km: 1.22 (1.16–1.29) µg/L, between 1.0 and less than 1.5 km: 1.18 (1.12–1.25) μg/L, between 1.5 and less than 3.0 km: 1.18 (1.11–1.25) μg/L, 3.0 km or more: 1.18 (1.09–1.27) μg/L, *p* = 0.641). There was no significant difference according to past mining work experience (with experience: 1.14 (1.07–1.22) μg/L, without experience: 1.21 (1.16–1.27) μg/L, *p* = 0.053).

### 3.4. Comparison of Urine Cadmium Concentration

Table 4 shows the comparison results of urine cadmium concentrations adjusted for sex, age, smoking status, drinking status, monthly household income, and year of survey.

The concentration of 1.25 (1.13–1.37) μg/g cr of AMS was found to be significantly higher than that of KNEHS, 0.68 (0.63–0.74) μg/g cr (*p* < 0.001).

In the case of AMS, women showed significantly higher concentrations than men (men: 0.89 (0.81–0.98) μg/g cr, women: 1.74 (1.55–1.94) μg/g cr, *p* < 0.001). There was a clear trend of increasing concentrations with age (19–39 years: 0.57 (0.42–0.76) μg/g cr, 40s: 1.05 (0.90–1.24) μg/g cr, 50s: 1.47 (1.34−1.62) μg/g cr, 60s: 1.80 (1.65–1.97) μg/g cr, over 70s: 1.89 (1.73–2.07) μg/g cr, *p* < 0.001). The distribution of concentrations by gender and age showed the same tendency in KNEHS, and there was a significant difference when compared with AMS by the level of factors. The urine cadmium concentrations in Honam region in AMS were highest, while those in the southern area, including Yeongnam, were relatively higher than those in the central area (Seoul, Gyeonggi, Gangwon: 0.84 (0.76–0.92) μg/g cr, Chungcheong: 1.17 (1.07–1.28) μg/g) cr, Honam: 1.56 (1.39–1.76) μg/g cr, Yeongnam: 1.33 (1.21–1.45) μg/g cr, *p* < 0.001). The regional concentrations of KNEHS also showed similar distribution patterns.

The cadmium urine concentrations were higher in the order current smoker, past smoker, and non-smoker (current smoker: 1.35 (1.20–1.52) μg/g cr, past smoker: 1.21 (1.08–1.36) μg/g cr, non-smoker: 1.18 (1.07)−1.31) μg/g cr, *p* = 0.025). According to the current drinking status, the highest cadmium concentration was observed in non-drinkers’ urine, and there was no characteristic tendency (current drinker: 1.24 (1.13–1.37) μg/g cr, former drinker: 1.20 (1.07–1.36) μg/g cr, non-drinker: 1.29 (1.16–1.43) μg/g cr, *p* = 0.263). The monthly household income and the cadmium concentrations in urine also showed no correlation (less than 1 million won: 1.27 (1.15–1.41)) μg/g cr, between 1 and less than 2 million won: 1.22 (1.09–1.37) μg/g cr, between 2 and less than 3 million won: 1.23 (1.07–1.42) μg/g cr, more than 3 million won: 1.25 (1.09–1.44) μg/g cr, *p* = 0.795). In the case of KNEHS, current smokers had the highest concentration and showed the same distribution as in AMS. In KNEHS, there was a significantly increasing cadmium urine concentration with a lower monthly household income, which differed from the AMS results. As the distance from the mine to the residential area increased, the concentration level decreased (less than 0.5 km: 1.17 (1.06–1.30) µg/g cr, between 0.5 and less than 1.0 km: 1.23 (1.12–1.35) µg/g cr, between 1.0 and less than 1.5 km: 1.25 (1.13–1.37) μg/g cr, between 1.5 and less than 3.0 km: 1.06 (0.95–1.18) μg/g cr, 3.0 km or more: 0.83 (0.72–0.95) μg/g cr, *p* < 0.001). There were no differences in concentration levels according to past mining experience (with experience: 1.21 (1.07–1.37) μg/g cr, without experience: 1.14 (1.05–1.23) μg/g cr, *p* = 0.260)

### 3.5. Proportion above Reference Value

As an indicator for the distribution of high concentrations of heavy metals in subjects in AMS, the excess subject proportion was calculated with reference to the 95th percentile value of the general population data obtained from KNHANES and KNEHS, and the results are presented in Table 5.

The 95th percentile of blood lead in KNHANES was 4.49 μg/dL, and the excess proportion was 6.23%, showing no pronounced difference in the proportion of high concentration subjects. The 4.52 μg/dL excess proportion of KNEHS was 6.12%. On the other hand, for blood cadmium, the excess proportion against the reference value 2.72 μg/L of the KNHANES standard was 12.89%, showing many high-concentration subjects in the AMS data. Similarly, the urine cadmium excess proportion against the reference value 2.48 μg/g cr of the KNEHS was 30.58%, confirming a high number of high-concentration subjects in the AMS data.

## 4. Discussion

This study evaluated exposure levels of blood lead, cadmium, urine cadmium, and arsenic for residents near abandoned metal mines, vulnerable regions in terms of environmental health, used national biomonitoring data to represent the general population, and compared the concentration levels between them.

### 4.1. Blood Lead Concentration

The concentration of blood lead adjusted for the demographic characteristics of AMS was 2.39 μg/dL, which was higher than that of KNHANES, 2.24 μg/dL, and of KNEHS, 1.92 μg/dL, to which simple random resampling was applied at the same proportion, according to sex and age distribution. For KNEHS, there was a significant difference in the concentration level. For AMS, the uniformity of subject characteristics during the time of survey was not secured, but there was a decreasing tendency in the annual exposure level (2013: 2.47 μg/dL, 2014: 2.22 μg/dL, 2015: 2.47 μg/dL 2016: 2.11 μg/dL, 2017: 2.13 μg/dL). KNHANES values also showed a clear decreasing exposure level tendency for the representative value estimation of the general population over 19 years in age in Korea (2005: 2.61 μg/dL, 2008: 2.32 μg/dL, 2009: 2.29 μg/dL, 2010: 2.21 μg/dL, 2011: 2.14 μg/dL, 2012: 2.00 μg/dL, 2013: 1.94 μg/dL, 2016: 1.76 μg/dL, 2017: 1.48 μg/dL) [6]. However, when compared with U.S. NHANES analysis results of the general population over 20 years (2005–2006: 1.41 μg/dL, 2007–2008: 1.38 μg/dL, 2009–2010: 1.23 μg/dL, 2011–2012: 1.19 μg/dL, 2013–2014: 0.97 μg/dL, 2015–2016: 0.92 μg/dL) [7], both the abandoned metal mines, the vulnerable regions, and the general population results for Korea are relatively higher in terms of the concentration level. On the other hand, the concentration of blood lead in a German Environmental Survey (GerES) III in 1998 was 3.16 μg/dL [8], which was somewhat high; however, the results presented in a recent study from 2011 [9] (men: 2.10 μg/dL, women: 1.35 μg/dL) were lower than those of AMS. In the AMS results, there was no value that exceeded 15 µg/dL, the human biomonitoring (HBM) I reference value from the German HBM commission; but as a correlation with adverse health effects was reported in low-level chronic exposure cases [10], the existing reference value was withdrawn [11]. In this regard, the need for management in the lower concentration level has been raised. In fact, The U.S. Center for Disease Control and Prevention (CDC) has suggested 5 µg/dL as a reference value [12], where 3.85% of AMS subjects exceeded this value, which was 3.04% when adjusted for sex and age. Furthermore, Gilbert et al. [13] proposed 2 μg/dL as a reference for an effective management of lead because there has been no threshold value for health effects. When compared against this reference value, AMS subjects showed an excess rate of 61.59% (adjusted for sex and age 59.02%). As a representative value of the general population, the weighted excess proportion of the KNHANES data in the seventh (2016, 2017) survey was estimated to be 30.38%, and similarly, the weighted excess rate of KNEHS in the third (2015–2017) survey was 31.08%. Bello et al. [14] assessed blood lead levels in adults and children in mining areas, with 14% of adults exceeding 5 μg/dL and reported a significant correlation with lead concentrations in water and soil. Similarly, a number of studies have suggested a lead contamination of soil and water quality by metal mines [15,16,17,18,19,20]. Excluding occupational exposure, drinking water, food, and inhaled air contaminated with lead may be the main sources of exposure [21]. A domestic study evaluated lead exposure through air, soil, drinking water, and food, and estimated the contribution rate of each medium to the lead exposure in adults aged 20–64 to be 86.79% for food intake, 7.34% for air, and 5.21% for soil [22]. Relatively high-concentration exposure levels in abandoned metal mines are presumed to be primarily attributable to ingested food produced from contaminated soil [2].

### 4.2. Blood Cadmium Concentration

The blood cadmium concentration in AMS was 1.19 μg/L, which was not significantly different from that of KNHANES, with 1.22 μg/L, whereas the urine cadmium concentration was 1.25 μg/g cr, which was higher than that of KNEHS, with 0.68 μg/g cr. These differences were statistically significant (*p* < 0.001). Blood cadmium is an indicator for relatively recent exposure levels [23,24,25,26], while urine cadmium is also responsive to recent exposure to some extent but is mainly used as a total cumulative indicator for long-term exposure [27]. Estimates of blood cadmium concentrations of the general population over 19 years in Korea from KNHANES data have been less than 1 μg/L since 2008 (2005: 1.52 μg/L, 2008: 0.93 μg/L, and 2009: 0.94 μg/L, 2010: 1.02 μg/L, 2011: 1.00 μg/L, 2012: 0.96 μg/L, 2013: 0.85 μg/L, 2016: 0.93 μg/L, 2017: 0.77 μg/L) [6]. The urine cadmium concentration of the KNEHS results were 0.6 μg/g cr or less (2009: 0.64 μg/g cr, 2010: 0.65 μg/g cr, 2011: 0.59 μg/g cr, 2012: 0.45 μg/g cr, 2013: 0.39 μg/g cr, 2014: 0.44 μg/g cr, 2015: 0.59 μg/g cr, 2016: 0.57 μg/g cr, 2017: 0.24 μg/g cr), and a significant difference in exposure levels was observed when comparing values from the same year. Both KNHANES and KNEHS did not employ simultaneous blood and urine analyses, which limits the inference on the cause of these differences. In the results of AMS, in which both blood and urine cadmium analyses were performed, each biomarker showed very similar exposure levels. The linear correlation coefficient (Pearson’s correlation coefficient) was 0.426, and international studies reported a similar correlation coefficient of 0.57 [28]. In the future, it is thought to be necessary to evaluate the difference and correlation between blood and urine biomarkers as representative values for blood and urine cadmium levels in the general population of Korea. On the other hand, U.S. NHANES results showed that urine cadmium was 0.2 μg/g cr or less (2005–2006: 0.24 μg/g cr, 2007–2008: 0.25 μg/g cr, 2009–2010: 0.24 μg/g cr, 2011–2012: 0.22 μg/g cr, 2013–2014: 0.18 μg/g cr, 2015–2016: 0.19 μg/g cr) [7], which is very low compared to the AMS and KNEHS results. A reference value for acute exposure of blood cadmium, 5 μg/L, was applied to AMS [29] and the excess proportion was 1.79%. As a result of the multiple biomonitoring of the general population, a reference value of concentration levels no more than 1 μg/L was presented [30,31,32,33]. In particular, Wilhelm et al. [34] suggested a value of 0.5 μg/L for children. The biomonitoring reference value (RV95) is defined as the 95th population percentile of the concentration of heavy metals and illustrates an exposure to environmental contaminants in the community. For AMS, the excess proportion of 1 μg/L was 73.85%, and the sex- and age-adjusted excess proportion was 63.01%. As a representative value for the general population, the weighted excess proportion of the seventh KNHANES results was estimated to be 42.06%. In the 95th percentile value comparison, the value of AMS was 3.80 μg/L, which is higher as compared to 2.73 μg/L of the KNHANES resampled samples. On the other hand, the weighted estimate of the seventh KNHANES results, which reflects the demographic distribution of the general population, was 2.22 µg/L, which is significantly different from the 1.35 µg/L of the 2015–2016 NHANES results. For urine cadmium, the occupational exposure limit of 5 μg/g cr was also applied to AMS [29]. The AMS excess proportion against the reference value was 6.86%. HBM I 1 μg/L and HBM II 4 μg/L were presented as reference values for the general adult population [11], and there were differences depending on the creatinine correction, but the excess proportions of AMS were 77.02% and 11.76%, respectively (sex- and age-adjusted proportions, 63.56%, 7.84%). While HBM I refers to concentration levels that have no health effects without requirements for further action, HBM II refers to concentration levels that increase the risk of adverse health effects and require active efforts to reduce exposure levels [11]. It is estimated that more than 10% of the residents of abandoned metal mines, vulnerable regions, need medical consultation and interventions for an exposure level reduction. The excess proportion against the 4 μg/L reference value of the resampled KNHANES sample was 1.01% and about 10 times higher or lower than the AMS result. Estimates of the general population using the third KNEHS survey results showed an excess proportion of 20.27% against the reference value of 1 μg/L and 0.43% against a value of 4 μg/L. According to the assessment of the general population of Korea, there are relatively few cases exceeding the HBM II reference value that require active countermeasures. Several studies have reported a cadmium contamination of soil, water quality, and food by metal mines [18,19,35,36,37], which can act as a major source of exposure [38]. In a domestic study on the exposure levels of cadmium separated by medium, which is somewhat different from the current study, depending on the secondary data used in the estimation, it was reported that 95% of the cadmium exposure of adults aged 20 to 64 was via ingested food. Atmospheric inhalation had a contribution of about 3% [39]. Relatively high-concentration exposure levels in abandoned metal mines are presumed to be mainly attributable to the ingested food produced from contaminated soil [2].

### 4.3. Exposure Assessment to Heavy Metals

The assessment of the heavy metal exposure level of abandoned metal mines in Korea revealed that the exposure levels were higher than those of the general population in Korea, and there was a significant difference in the general population of Korea, including abandoned metal mines, when the results were compared to the exposure levels reported overseas. Examining the excess proportion of blood lead, since 2015, the excess proportion against 5 μg/dL for the general domestic and oversea populations was 0.1% or less, whereas for AMS, it was 3.0% or higher, indicating the requirement for more active efforts for management. In addition, for urine cadmium, in comparison to the excess proportion HBM II of 4 μg/L for the general domestic and overseas population of 0.43%, that of AMS was 10% or higher, indicating a significant difference. There is more pressing need for cadmium exposure level management, and intervention measures for an exposure level reduction should be taken for those who exceed the reference value. 

### 4.4. Strengths and Limitations of the Study

In this study, heavy metal exposure levels were assessed for residents in areas of abandoned metal mines with a high risk of environmental pollution and adverse health effects. Detailed inferences were made by direct comparison with the general population using national biomonitoring data. In particular, samples were constructed considering demographic characteristics to minimize bias and ensure validity by presenting comparable estimates of exposure levels and excess proportions against various reference values. However, an absence of correlations with environmental factors in assessing the exposure levels of hazardous substances in vulnerable regions can be pointed out as a limitation of this study. In the case of AMS, a limited environmental pollution assessment using secondary data has been carried out in this study; however, it is difficult to generate data with sufficiently convincing evidence in the relatively high-concentration exposure status that is presented in this study. In the future, it is necessary to evaluate the influence of environmental pollution on the various impact factors of heavy metal exposure levels for residents in abandoned metal mines.

## 5. Conclusions

Compared to the general population, the concentrations of lead and cadmium in the residents of abandoned metal mines, i.e., vulnerable regions, were found to be higher. As for the exposure pathways of these heavy metals, crop intake, groundwater intake, soil contact, and fugitive dust intake can be taken into account, and monitoring and management at the individual and national level should be conducted accordingly.

## Figures and Tables

**Table 1:** Distribution of the blood lead (Pb), cadmium (Cd), urine Cd, and arsenic (As) concentration.

Data	Heavy Metal	n	GM (95% CI)	Median (Range)	P75	P90	P95	P99
Second AMS (2013–2017)	Blood Pb (μg/dL)	4463	2.28 (2.25–2.31)	2.25 (0.49–21.99)	3.01	3.93	4.72	6.95
	Blood Cd (μg/L)	4467	1.43 (1.40–1.45)	1.40 (0.14–17.95)	2.10	3.00	3.80	6.03
	Urine Cd (μg/g cr)	2433	1.67 (1.62–1.73)	1.74 (0.01–38.34)	2.82	4.29	5.81	9.68
	Urine As (μg/L)	3813	30.32 (29.50–31.17)	31.17 (0.33–855.23)	53.90	86.11	117.10	213.56
KNHANES (2008–2013, 2016, 2017)	Blood Pb (μg/dL)	1815	2.21 (2.16–2.25)	2.24 (0.51–26.51)	2.92	3.77	4.49	6.23
	Blood Cd (μg/L)	1815	1.24 (1.22–1.27)	1.25 (0.05–11.05)	1.71	2.30	2.73	3.77
KNEHS (2009–2017)	Blood Pb (μg/dL)	2319	2.11 (2.07–2.16)	2.14 (0.21–22.58)	2.87	3.76	4.52	7.10
	Urine Cd (μg/g cr)	1972	0.82 (0.80–0.85)	0.83 (0.03–15.66)	1.31	1.91	2.49	4.04
	Urine As (μg/L)	561	42.09 (39.65–44.68)	43.60 (2.00–568.90)	68.50	97.80	135.20	187.00

n: sample size; GM (95% CI): geometric mean (95% confidence interval); P: percentile; AMS: abandoned metal mines survey; KNHANES: Korea National Health and Nutrition Examination Survey; KNEHS: Korea National Environmental Health Survey.

**Table 2:** Adjusted geometric means of the blood lead (Pb) concentration (μg/dL).

Factor	Second AMS (2013–2017)				KNHANES (2008–2013, 2016, 2017)				KNEHS (2009–2017)				*p*-Value
	n	Adjusted GM (95% CI)	c	r	n	Adjusted GM (95% CI)	c	r	n	Adjusted GM (95% CI)	c	r	
**Total**	4463	2.39 (2.30–2.48)		^a^	1815	2.24 (2.14–2.35)		^a^	2319	1.92 (1.83–2.00)		^b^	<0.001
Sex													
Male	1752	2.64 (2.54–2.74)		^a^	713	2.47 (2.34–2.61)		^a^	896	2.16 (2.06–2.26)		^b^	<0.001
Female	2711	2.16 (2.07–2.25)		^a^	1102	2.04 (1.92–2.16)		^a^	1423	1.70 (1.61–1.80)		^b^	<0.001
*p*-value		<0.001				<0.001				<0.001			
Age (year)													
30–39	53	1.88 (1.68–2.11)	^b^	^a^	22	1.96 (1.64–2.34)		^a^	26	1.29 (1.10–1.52)	^b^	^a^	<0.001
40–49	174	2.38 (2.23–2.54)	^ab^	^a^	71	2.16 (1.96–2.39)		^ab^	89	1.79 (1.63–1.96)	^ab^	^b^	<0.001
50–59	702	2.68 (2.58–2.78)	^a^	^a^	285	2.37 (2.24–2.50)		^ab^	381	2.27 (2.16–2.38)	^a^	^b^	<0.001
60–69	1183	2.61 (2.52–2.71)	^a^	^a^	480	2.41 (2.30–2.51)		^ab^	645	2.23 (2.14–2.31)	^a^	^b^	<0.001
≥70	2351	2.47 (2.38–2.56)	^ab^	^a^	957	2.36 (2.28–2.45)		^a^	1178	2.21 (2.13–2.29)	^a^	^a^	<0.001
*p*-value		<0.001				0.082				<0.001			
Residence area													
Seoul, Gyeonggi, Gangwon	896	2.74 (2.62–2.86)	^a^	^a^	853	2.18 (2.08–2.29)	^ab^	^b^	212	1.95 (1.78–2.13)	^a^	^b^	<0.001
Chungcheong	1403	2.55 (2.45–2.66)	^a^	^a^	238	2.31 (2.17–2.47)	^ab^	^ab^	89	1.78 (1.57–2.01)	^a^	^b^	<0.001
Honam	612	2.27 (2.16–2.38)	^b^	^a^	216	2.48 (2.32–2.66)	^a^	^a^	63	2.10 (1.85–2.39)	^a^	^a^	0.028
Yeongnam	1552	2.25 (2.16–2.35)	^b^	^a^	508	2.08 (1.96–2.19)	^b^	^ab^	198	1.71 (1.55–1.88)	^a^	^b^	<0.001
*p*-value		<0.001				<0.001				<0.001			
Smoking status													
Current smoker	568	2.54 (2.42–2.66)	^a^	^a^	249	2.45 (2.29–2.62)	^a^	^ab^	297	2.05 (1.93–2.18)	^a^	^b^	<0.001
Past smoker	729	2.38 (2.27–2.50)	^ab^	^a^	420	2.23 (2.09–2.38)	^ab^	^a^	447	1.87 (1.76–1.98)	^a^	^b^	<0.001
Never smoked	3166	2.25 (2.17–2.34)	^b^	^a^	1146	2.07 (1.96–2.18)	^b^	^ab^	1575	1.84 (1.75–1.93)	^a^	^b^	<0.001
*p*-value		<0.001				<0.001				0.002			
Drinking status													
Currently	1724	2.56 (2.47–2.66)	^a^	^a^	1015	2.39 (2.28–2.50)	^a^	^a^	1040	2.04 (1.96–2.14)	^a^	^b^	<0.001
Former	460	2.31 (2.19–2.43)	^b^	^a^	346	2.20 (2.07–2.34)	^a^	^ab^	224	1.87 (1.75–2.00)	^ab^	^b^	<0.001
Never	2279	2.30 (2.21–2.39)	^b^	^a^	454	2.16 (2.03–2.30)	^a^	^ab^	1055	1.84 (1.75–1.94)	^b^	^b^	<0.001
*p*-value		<0.001				<0.001				<0.001			
Month income (₩10,000)													
<100	3420	2.37 (2.29–2.46)	^a^	^a^	681	2.33 (2.20–2.47)		^a^	955	1.93 (1.83–2.03)		^b^	<0.001
100−<200	596	2.45 (2.34–2.57)	^a^	^a^	372	2.26 (2.12–2.40)		^ab^	487	1.94 (1.84–2.06)		^b^	<0.001
200−<300	229	2.26 (2.12–2.40)	^a^	^a^	245	2.21 (2.07–2.37)		^a^	335	1.92 (1.81–2.04)		^a^	<0.001
≥300	218	2.47 (2.32–2.63)	^a^	^a^	517	2.18 (2.07–2.30)		^ab^	542	1.87 (1.77–1.97)		^b^	<0.001
*p*-value		0.034				0.109				0.488			
Distance from mine (km)													
<0.5	697	2.29 (2.19–2.39)	^a^										
0.5−<1.0	1181	2.29 (2.20–2.38)	^a^										
1.0−<1.5	982	2.22 (2.13–2.31)	^a^										
1.5−<3.0	644	2.30 (2.21–2.41)	^a^										
≥3.0	325	2.13 (2.02–2.25)	^a^										
*p*-value		0.011											
Mining work history													
Yes	569	2.31 (2.21–2.42)											
No	3894	2.26 (2.19–2.34)											
*p*-value		0.249											

n: sample size; GM (95% CI): geometric mean (95% confidence interval); AMS: abandoned metal mines survey; KNHANES: Korea National Health and Nutrition Examination Survey; KNEHS: Korea National Environmental Health Survey; Adjusted GM: least square means (geometric mean) by sex, age, smoking status, drinking status, monthly income, year, c: post hoc grouping for column factor, r: post hoc grouping for row factor, ^a, b^: Bonferroni post hoc; estimates with the same letter are not significantly different.

**Table 3:** Adjusted geometric means of the blood cadmium (Cd) concentration (μg/L).

Factor	Second AMS (2013–2017)				KNHANES (2008–2013, 2016, 2017)				*p*-Value
	n	Adjusted GM (95% CI)	c	r	n	Adjusted GM (95% CI)	c	r	
**Total**	4467	1.19 (1.13–1.25)			1815	1.22 (1.15–1.29)			0.564
Sex									
Male	1752	0.98 (0.94–1.03)	^b^		713	1.01 (0.94–1.08)	^b^		0.591
Female	2715	1.43 (1.35–1.52)	^a^		1102	1.47 (1.37–1.58)	^a^		0.627
*p*-value		<0.001				<0.001			
Age (year)									
30–39	53	0.79 (0.69–0.92)	^e^		22	0.85 (0.68–1.06)	^d^		0.606
40–49	173	1.14 (1.05–1.24)	^c^		71	1.17 (1.03–1.33)	^ad^		0.768
50–59	702	1.30 (1.23–1.36)	^bc^		285	1.37 (1.28–1.46)	^a^		0.237
60–69	1183	1.38 (1.31–1.45)	^ab^		480	1.37 (1.30–1.45)	^a^		0.898
≥70	2356	1.46 (1.39–1.53)	^a^		957	1.43 (1.36–1.49)	^a^		0.485
*p*-value		<0.001				<0.001			
Residence area									
Seoul, Gyeonggi, Gangwon	896	0.93 (0.88–0.99)	^d^	^b^	853	1.15 (1.08–1.23)	^b^	^a^	<0.001
Chungcheong	1403	1.19 (1.13–1.25)	^b^		238	1.17 (1.08–1.27)	^ab^		0.707
Honam	612	1.20 (1.13–1.28)	^b^		216	1.33 (1.22–1.45)	^ab^		0.061
Yeongnam	1556	1.48 (1.40–1.56)	^a^		508	1.30 (1.21–1.39)	^a^		0.004
*p*-value		<0.001				<0.001			
Smoking status									
Current smoker	568	1.42 (1.34–1.52)	^a^		249	1.56 (1.43–1.69)	^a^		0.107
Past smoker	729	1.13 (1.06–1.20)	^b^		420	1.15 (1.06–1.24)	^b^		0.776
Never smoked	3170	1.04 (0.99–1.10)	^b^		1146	1.01 (0.95–1.08)	^b^		0.455
*p*-value		<0.001				<0.001			
Drinking status									
Currently	1725	1.20 (1.14–1.26)			1015	1.17 (1.10–1.24)	^a^		0.438
Former	459	1.14 (1.07–1.22)			346	1.27 (1.18–1.38)	^a^		0.041
Never	2283	1.22 (1.16–1.29)			454	1.21 (1.12–1.31)	^a^		0.923
*p*-value		0.077				0.024			
Month income (₩10,000)									
<100	3425	1.25 (1.19–1.31)	^a^		681	1.27 (1.18–1.36)			0.711
100–<200	595	1.17 (1.11–1.25)	^a^		372	1.20 (1.11–1.29)			0.726
200–<300	229	1.16 (1.07–1.25)	^a^		245	1.22 (1.12–1.33)			0.357
≥300	218	1.18 (1.09–1.27)	^a^		517	1.18 (1.11–1.26)			0.927
*p*-value		0.025				0.156			
Distance from mine (km)									
<0.5	697	1.20 (1.13–1.27)							
0.5–<1.0	1181	1.22 (1.16–1.29)							
1.0–<1.5	986	1.18 (1.12–1.25)							
1.5–<3.0	644	1.18 (1.11–1.25)							
≥3.0	325	1.18 (1.09–1.27)							
*p*-value		0.641							
Mining work history									
Yes	570	1.14 (1.07–1.22)							
No	3897	1.21 (1.16–1.27)							
*p*-value		0.053							

n: sample size; GM (95% CI): geometric mean (95% confidence interval); AMS: abandoned metal mines survey; KNHANES: Korea National Health and Nutrition Examination Survey; Adjusted GM: least square means (geometric mean) by sex, age, smoking status, drinking status, monthly income, year, c: post hoc grouping for column factor, r: post hoc grouping for row factor, ^a, b, c, d^: Bonferroni post hoc; estimates with the same letter are not significantly different.

**Table 4:** Adjusted geometric means of the urine cadmium (Cd) concentration (μg/g cr).

Factor	Second AMS (Years 2015–2017)				KNEHS (Years 2009–2017)				*p*-Value
	n	Adjusted GM (95% CI)	c	r	n	Adjusted GM (95% CI)	c	r	
**Total**	2433	1.25 (1.13–1.37)		^a^	1972	0.68 (0.63–0.74)		^b^	<0.001
Sex									
Male	998	0.89 (0.81–0.98)	^b^	^a^	827	0.55 (0.50–0.60)	^b^	^b^	<0.001
Female	1435	1.74 (1.55–1.94)	^a^	^a^	1145	0.86 (0.78–0.94)	^a^	^b^	<0.001
*p*-value		<0.001				<0.001			
Age (year)									
30–39	21	0.57 (0.42–0.76)	^d^		19	0.41 (0.30–0.56)	^c^		0.145
40–49	81	1.05 (0.90–1.24)	^cd^	^a^	80	0.62 (0.53–0.73)	^c^	^b^	<0.001
50–59	361	1.47 (1.34–1.62)	^c^	^a^	324	0.73 (0.67–0.80)	^ac^	^b^	<0.001
60–69	681	1.80 (1.65–1.97)	^a^	^a^	553	0.89 (0.84–0.96)	^a^	^b^	<0.001
≥70	1289	1.89 (1.73–2.07)	^a^	^a^	996	0.88 (0.83–0.94)	^a^	^b^	<0.001
*p*-value		<0.001				<0.001			
Residence area *									
Seoul, Gyeonggi, Gangwon	596	0.84 (0.76–0.92)	^d^		195	0.75 (0.64–0.87)	^a^		0.210
Chungcheong	867	1.17 (1.07–1.28)	^b^	^a^	79	0.80 (0.65–1.00)	^a^	^a^	0.001
Honam	212	1.56 (1.39–1.76)	^a^	^a^	56	1.06 (0.84–1.33)	^a^	^a^	0.003
Yeongnam	758	1.33 (1.21–1.45)	^ab^	^a^	169	0.90 (0.76–1.07)	^a^	^b^	<0.001
*p*-value		<0.001				0.003			
Smoking status									
Current smoker	320	1.35 (1.20–1.52)	^a^	^a^	264	0.75 (0.67–0.83)	^a^	^b^	<0.001
Past smoker	467	1.21 (1.08–1.36)	^a^	^a^	411	0.68 (0.61–0.75)	^a^	^b^	<0.001
Never smoked	1646	1.18 (1.07–1.31)	^a^	^a^	1297	0.63 (0.58–0.69)	^a^	^b^	<0.001
*p*-value		0.025				0.013			
Drinking status									
Currently	987	1.24 (1.13–1.37)		^a^	916	0.65 (0.60–0.71)		^b^	<0.001
Former	287	1.20 (1.07–1.36)		^a^	196	0.71 (0.63–0.80)		^b^	<0.001
Never	1159	1.29 (1.16–1.43)		^a^	860	0.69 (0.63–0.76)		^b^	<0.001
*p*-value		0.263				0.108			
Month income (₩10,000)									
<100	1771	1.27 (1.15–1.41)		^a^	782	0.73 (0.66–0.80)		^b^	<0.001
100–<200	380	1.22 (1.09–1.37)		^a^	414	0.67 (0.61–0.74)		^b^	<0.001
200–<300	141	1.23 (1.07–1.42)		^a^	295	0.65 (0.59–0.73)		^b^	<0.001
≥300	141	1.25 (1.09–1.44)		^a^	481	0.68 (0.62–0.75)		^b^	<0.001
*p*-value		0.795				0.090			
Distance from mine(km)									
< 0.5	406	1.17 (1.06–1.30)	^ac^						
0.5–<1.0	695	1.23 (1.12–1.35)	^a^						
1.0–<1.5	564	1.25 (1.13–1.37)	^a^						
1.5–<3.0	308	1.06 (0.95–1.18)	^c^						
≥3.0	124	0.83 (0.72–0.95)	^e^						
*p*-value		<0.001							
Mining work history									
Yes	194	1.21 (1.07–1.37)							
No	2239	1.14 (1.05–1.23)							
*p*-value		0.26							

n: sample size; GM (95% CI): geometric mean (95% confidence interval); AMS: abandoned metal mines survey; KNEHS: Korea National Environmental Health Survey; Adjusted GM: least square means (geometric mean) by sex, age, smoking status, drinking status, monthly income, year, c: post hoc grouping for column factor, r: post hoc grouping for row factor, ^a, b, c, d^: Bonferroni post hoc; estimates with the same letter are not significantly different, ^*^: adjusted by sex, age, smoking status, drinking status, month income.

**Table 5:** Proportion of excess subjects with respect to heavy metal concentrations.

Heavy Metal	Reference Value		Survey n (Denominator)	Excess	
				n (Numerator)	%
Blood Pb	KNHANES P95	4.49 μg/dL	4463	278	6.23
	KNEHS P95	4.52 μg/dL		273	6.12
	AMS	Female, age ≤ 45 yrs: 15 μg/dL, Others: 25 μg/dL		0	0
Blood Cd	KNHANES P95	2.72 μg/L	4467	576	12.89
	AMS	5.00 μg/L		80	1.79
Urine Cd	KNEHS P95	2.56 μg/g cr	2433	744	30.58
	ASM	Age ≤ 25 yrs: 3 μg/g cr, Others: 5 μg/g cr		167	6.86

Pd: lead; Cd: cadmium; P: percentile; AMS: abandoned metal mines survey; KNHANES: Korea National Health and Nutrition Examination Survey; KNEHS: Korea National Environmental Health Survey; Survey n: total number of subjects in the AMS; Excess n: the number of subjects exceeding the reference value among total subjects in the AMS.
